# 2,2′-Diethoxy-4,4′-[(*E*,*E*)-hydrazine­diyl­idenebis(methanylylidene)]diphenol

**DOI:** 10.1107/S1600536812038354

**Published:** 2012-09-22

**Authors:** Wisam Naji Atiyah Al-Mehana, Rosiyah Yahya, Faridah Sonsudin, Ihsan Naji Atiyah Al-Mehana, Kong Mun Lo

**Affiliations:** a Department of Chemistry, University of Malaya, 50603 Kuala Lumpur, Malaysia; b Centre for Foundation Studies, University of Malaya, 50603 Kuala Lumpur, Malaysia; c Nursing Department, Kufa Technical Institute, PO Box 49, Kufa/Najaf, Iraq

## Abstract

The complete molecule of the title compound, C_18_H_20_N_2_O_4_, is generated by inversion symmetry. The conformation around the C=N bond is *E*. With the exception of the eth­oxy substituent, the mol­ecule is essentially planar with an r.m.s. deviation of 0.0455 Å. In the crystal, mol­ecules are linked by O—H⋯N hydrogen bonds into a two-dimensional supra­molecular network parallel to the *bc* plane.

## Related literature

For the structure of 4,4′-(1*E*,1′*E*)-1,2-diylidenebis(methan-1-yl-1-yl­idene) bis­(2-meth­oxy­phenol), see: Qu *et al.* (2005 ▶). For applications of azines and their derivatives, see: Dudis *et al.* (1993 ▶); Facchetti *et al.* (2002 ▶); Kim *et al.* (2010 ▶); Pandeya *et al.* (1999 ▶); Wadher *et al.* (2009 ▶).
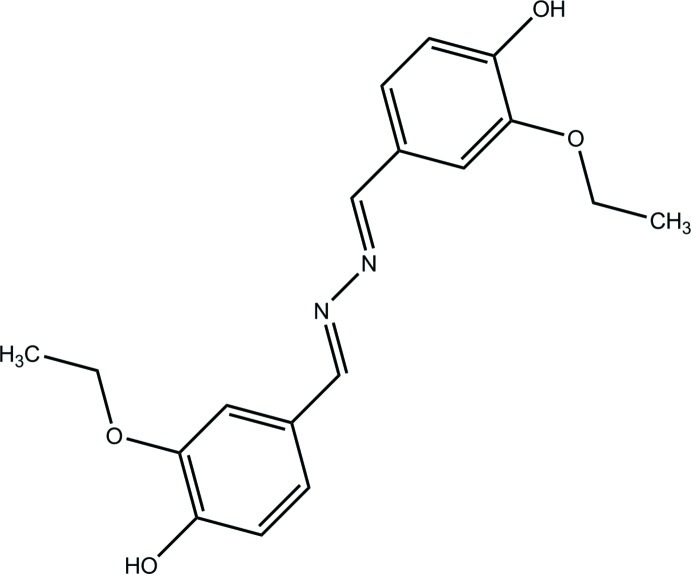


## Experimental

### 

#### Crystal data

C_18_H_20_N_2_O_4_
*M* *_r_* = 328.36
Monoclinic, 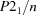

*a* = 5.2176 (1) Å
*b* = 10.3422 (1) Å
*c* = 14.9135 (2) Å
β = 97.206 (1)°
*V* = 798.40 (2) Å^3^
*Z* = 2
Mo *K*α radiation
μ = 0.10 mm^−1^
*T* = 100 K
0.16 × 0.08 × 0.08 mm

#### Data collection

Bruker APEXII CCD area-detector diffractometer
Absorption correction: multi-scan (*SADABS*; Sheldrick, 1996) ▶ *T*_min_ = 0.650, *T*_max_ = 0.746
7447 measured reflections
1831 independent reflections
1654 reflections with *I* > 2σ(*I*)
*R*_int_ = 0.020

#### Refinement

*R*[*F* ^2^ > 2σ(*F* ^2^)] = 0.034
*wR*(*F* ^2^) = 0.098
*S* = 1.05
1831 reflections
111 parameters
H-atom parameters constrained
Δρ_max_ = 0.33 e Å^−3^
Δρ_min_ = −0.23 e Å^−3^

### 

Data collection: *APEX2* (Bruker, 2009 ▶); cell refinement: *SAINT* (Bruker, 2009 ▶); data reduction: *SAINT*; program(s) used to solve structure: *SHELXS97* (Sheldrick, 2008 ▶); program(s) used to refine structure: *SHELXL97* (Sheldrick, 2008 ▶); molecular graphics: *X-SEED* (Barbour, 2001 ▶); software used to prepare material for publication: *publCIF* (Westrip, 2010 ▶).

## Supplementary Material

Crystal structure: contains datablock(s) I, global. DOI: 10.1107/S1600536812038354/zj2092sup1.cif

Structure factors: contains datablock(s) I. DOI: 10.1107/S1600536812038354/zj2092Isup2.hkl

Additional supplementary materials:  crystallographic information; 3D view; checkCIF report

## Figures and Tables

**Table 1:** Hydrogen-bond geometry (Å, °)

*D*—H⋯*A*	*D*—H	H⋯*A*	*D*⋯*A*	*D*—H⋯*A*
O2—H2⋯N1^i^	0.84	1.99	2.7787 (12)	156

Symmetry code: (i) 
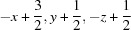
.

**Fig. 1.**
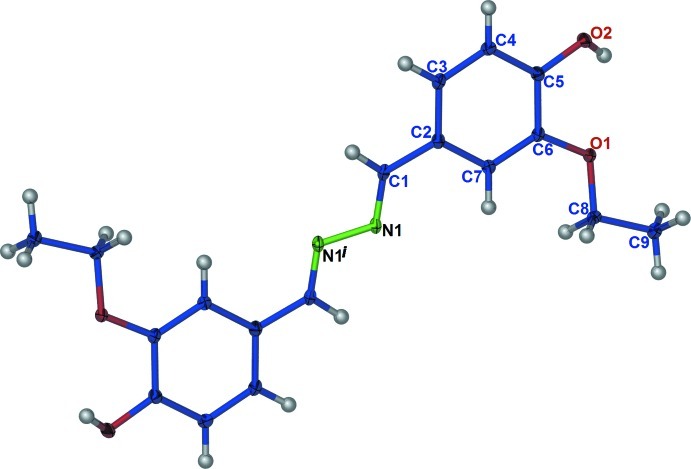
The molecular structure of 4,4'-(1E,1'E)-1,2-diylidenebis(methan-1-yl-1-ylidene) bis(2-ethoxyphenol) showing 50% probability displacement ellipsoids. Hydrogen atoms are drawn as spheres of arbitrary radius. Symmetry operation i: -x + 2, -y + 1, -z + 1.

**Fig. 2.**
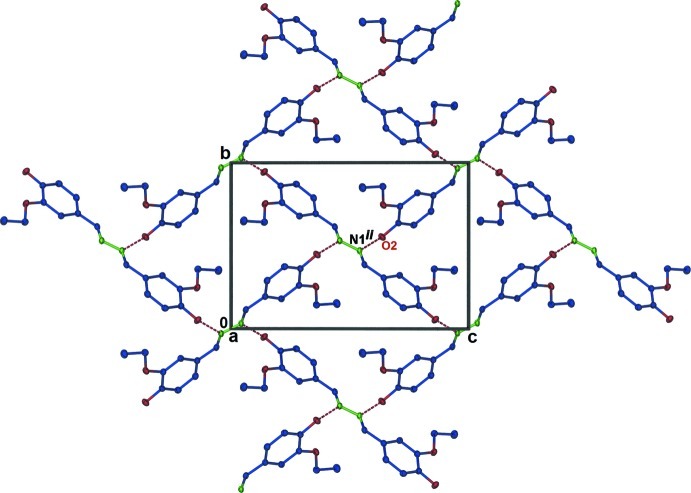
A view of the two-dimensional supramolecular network in the title compound showing the O—H···N hydrogen bonds (in red dotted lines).

## supplementary crystallographic information

### Comment

Aromatic carbonyl compounds react easily with hydrazine forming hydrazones, which could condense with a second molecule of the carbonyl compound to yield an azine. Due to the fascinating physical and chemical properties, azines and their derivatives have been extensively applied in such area as dyes [Kim *et al.*], non-linear fluorophores [Facchetti *et al.*], biological and pharmaceutical applications [Wadher *et al.*, Pandeya *et al.*]. Furthermore, there are many reports on polyazines as highly conjugated polymers in electronic, optoelectronic and photonic applications [Dudis *et al*]. In our work on a new class of monomers based upon the hydrazone moieties, we report here a new bis imine monomer. The title compound, C_18_H_20_N_2_O_4_, is centrosymmetric around the central azine bond [N1—N1^i^ = 1.416 (2) Å; symmetry operation i: -*x* + 2, -*y* + 1, -*z* + 1], with the *E* configuration around the N1=C1 bond [1.284 (1) Å]. In the crystal structure of the title compound in Fig 2, the molecules are linked together by O–H···N hydrogen bonds [O2—H2···N1^ii^ = 2.7782 (12) Å; symmetry operation ii: 3/2 - *x*, 1/2 + *y*, 1/2 + *z*] resulting in the formation of a two-dimensional supramolecular network which propagated parallel to the *bc* plane. C—H···pi interaction is also present; C8—H8b···*Cg1*^iii^ = 2.71 Å where *Cg1* is the centroid of the ring C2 - C7, [symmetry code: (iii) -1 + *x*, *y*, *z*].In contrast to the title compound, the methoxy substituted analogue [Qu, *et al.*] consists of two asymmetric units with the presence of additional intermolecular O—H..O hydrogen bonds with the adjacent asymmetric unit.

### Experimental

A mixture of 3-ethoxy-4-hydroxybenzaldehyde (3 g, 18 mmol), hydrazine sulfate (1.17 g, 9 mmol) and 1.7 ml of concentrated ammonia solution in 20 ml of 95% ethanol was stirred for 3 h. The solvent was removed under reduced pressure and the yellow residue was recrystallized from tetrahydrofuran to yield yellow crystals, m.p. 471 - 472 K.

### Refinement

Hydrogen atoms were placed at calculated positions (C–H 0.95 to 0.99 Å and O–H 0.84 Å) and were treated as riding on their parent carbon atoms, with *U*(H) set to 1.2–1.5 times *U*_eq_(C).

### Crystal data

**Table:**

C_18_H_20_N_2_O_4_	*F*(000) = 348
*M**_r_* = 328.36	*D*_x_ = 1.366 Mg m^−^^3^
Monoclinic, *P*2_1_/*n*	Melting point = 471–472 K
Hall symbol: -P 2yn	Mo *K*α radiation, λ = 0.71073 Å
*a* = 5.2176 (1) Å	Cell parameters from 4187 reflections
*b* = 10.3422 (1) Å	θ = 2.4–28.4°
*c* = 14.9135 (2) Å	µ = 0.10 mm^−^^1^
β = 97.206 (1)°	*T* = 100 K
*V* = 798.40 (2) Å^3^	Block, yellow
*Z* = 2	0.16 × 0.08 × 0.08 mm

### Data collection

**Table:**

Bruker APEXII CCD area-detector diffractometer	1831 independent reflections
Radiation source: fine-focus sealed tube	1654 reflections with *I* > 2σ(*I*)
Graphite monochromator	*R*_int_ = 0.020
ω scans	θ_max_ = 27.5°, θ_min_ = 2.4°
Absorption correction: multi-scan (*SADABS*; Sheldrick, 1996)	*h* = −6→6
*T*_min_ = 0.650, *T*_max_ = 0.746	*k* = −13→13
7447 measured reflections	*l* = −19→18

### Refinement

**Table:**

Refinement on *F*^2^	Primary atom site location: structure-invariant direct methods
Least-squares matrix: full	Secondary atom site location: difference Fourier map
*R*[*F*^2^ > 2σ(*F*^2^)] = 0.034	Hydrogen site location: inferred from neighbouring sites
*wR*(*F*^2^) = 0.098	H-atom parameters constrained
*S* = 1.05	*w* = 1/[σ^2^(*F*_o_^2^) + (0.052*P*)^2^ + 0.3158*P*] where *P* = (*F*_o_^2^ + 2*F*_c_^2^)/3
1831 reflections	(Δ/σ)_max_ < 0.001
111 parameters	Δρ_max_ = 0.33 e Å^−^^3^
0 restraints	Δρ_min_ = −0.23 e Å^−^^3^

### Special details

**Table:**

Geometry. All e.s.d.'s (except the e.s.d. in the dihedral angle between two l.s. planes) are estimated using the full covariance matrix. The cell e.s.d.'s are taken into account individually in the estimation of e.s.d.'s in distances, angles and torsion angles; correlations between e.s.d.'s in cell parameters are only used when they are defined by crystal symmetry. An approximate (isotropic) treatment of cell e.s.d.'s is used for estimating e.s.d.'s involving l.s. planes.
Refinement. Refinement of *F*^2^ against ALL reflections. The weighted *R*-factor *wR* and goodness of fit *S* are based on *F*^2^, conventional *R*-factors *R* are based on *F*, with *F* set to zero for negative *F*^2^. The threshold expression of *F*^2^ > σ(*F*^2^) is used only for calculating *R*-factors(gt) *etc*. and is not relevant to the choice of reflections for refinement. *R*-factors based on *F*^2^ are statistically about twice as large as those based on *F*, and *R*- factors based on ALL data will be even larger.

### Fractional atomic coordinates and isotropic or equivalent isotropic displacement parameters (Å^2^)

**Table:**

	*x*	*y*	*z*	*U*_iso_*/*U*_eq_	
C1	1.1226 (2)	0.61425 (10)	0.43730 (7)	0.0140 (2)	
H1	1.2770	0.6254	0.4776	0.017*	
C2	1.0867 (2)	0.69416 (10)	0.35602 (7)	0.0138 (2)	
C3	1.2699 (2)	0.79013 (10)	0.34702 (7)	0.0150 (2)	
H3	1.4163	0.7982	0.3914	0.018*	
C4	1.2397 (2)	0.87397 (10)	0.27353 (7)	0.0149 (2)	
H4	1.3647	0.9394	0.2683	0.018*	
C5	1.0277 (2)	0.86239 (10)	0.20780 (7)	0.0137 (2)	
C6	0.84916 (19)	0.76106 (10)	0.21383 (7)	0.0133 (2)	
C7	0.87615 (19)	0.67949 (10)	0.28809 (7)	0.0140 (2)	
H7	0.7521	0.6135	0.2931	0.017*	
C8	0.4920 (2)	0.64171 (10)	0.14038 (7)	0.0163 (2)	
H8B	0.3966	0.6416	0.1937	0.020*	
H8A	0.5954	0.5614	0.1412	0.020*	
C9	0.3053 (2)	0.64918 (12)	0.05452 (8)	0.0217 (3)	
H9A	0.2111	0.7313	0.0529	0.033*	
H9B	0.1825	0.5772	0.0528	0.033*	
H9C	0.4011	0.6439	0.0022	0.033*	
N1	0.95919 (17)	0.52993 (9)	0.45792 (6)	0.0138 (2)	
O1	0.65817 (14)	0.75311 (7)	0.14250 (5)	0.0160 (2)	
O2	1.00276 (14)	0.94690 (7)	0.13780 (5)	0.01629 (19)	
H2	0.8462	0.9532	0.1167	0.024*	

### Atomic displacement parameters (Å^2^)

**Table:**

	*U*^11^	*U*^22^	*U*^33^	*U*^12^	*U*^13^	*U*^23^
C1	0.0148 (5)	0.0157 (5)	0.0109 (5)	0.0022 (4)	−0.0010 (3)	−0.0018 (4)
C2	0.0150 (5)	0.0141 (5)	0.0119 (5)	0.0026 (4)	0.0011 (4)	−0.0007 (4)
C3	0.0139 (5)	0.0169 (5)	0.0136 (5)	0.0009 (4)	−0.0013 (4)	−0.0016 (4)
C4	0.0143 (5)	0.0141 (5)	0.0162 (5)	−0.0011 (4)	0.0012 (4)	−0.0007 (4)
C5	0.0155 (5)	0.0131 (5)	0.0127 (5)	0.0020 (4)	0.0025 (4)	0.0007 (4)
C6	0.0125 (5)	0.0146 (5)	0.0123 (5)	0.0010 (4)	−0.0004 (4)	−0.0009 (4)
C7	0.0147 (5)	0.0139 (5)	0.0134 (5)	−0.0002 (4)	0.0012 (4)	0.0004 (4)
C8	0.0168 (5)	0.0154 (5)	0.0156 (5)	−0.0036 (4)	−0.0017 (4)	0.0009 (4)
C9	0.0225 (6)	0.0236 (6)	0.0172 (5)	−0.0062 (4)	−0.0043 (4)	0.0018 (4)
N1	0.0162 (4)	0.0148 (4)	0.0098 (4)	0.0031 (3)	−0.0008 (3)	0.0004 (3)
O1	0.0162 (4)	0.0167 (4)	0.0135 (4)	−0.0036 (3)	−0.0039 (3)	0.0033 (3)
O2	0.0144 (4)	0.0169 (4)	0.0167 (4)	−0.0009 (3)	−0.0014 (3)	0.0052 (3)

### Geometric parameters (Å, º)

**Table:**

C1—N1	1.2835 (14)	C6—C7	1.3853 (14)
C1—C2	1.4596 (14)	C7—H7	0.9500
C1—H1	0.9500	C8—O1	1.4399 (12)
C2—C3	1.3959 (15)	C8—C9	1.5106 (14)
C2—C7	1.4062 (14)	C8—H8B	0.9900
C3—C4	1.3910 (15)	C8—H8A	0.9900
C3—H3	0.9500	C9—H9A	0.9800
C4—C5	1.3880 (14)	C9—H9B	0.9800
C4—H4	0.9500	C9—H9C	0.9800
C5—O2	1.3551 (12)	N1—N1^i^	1.4163 (16)
C5—C6	1.4126 (14)	O2—H2	0.8400
C6—O1	1.3658 (12)		
			
N1—C1—C2	124.33 (9)	C6—C7—C2	120.14 (9)
N1—C1—H1	117.8	C6—C7—H7	119.9
C2—C1—H1	117.8	C2—C7—H7	119.9
C3—C2—C7	119.34 (9)	O1—C8—C9	107.47 (8)
C3—C2—C1	117.68 (9)	O1—C8—H8B	110.2
C7—C2—C1	122.98 (9)	C9—C8—H8B	110.2
C4—C3—C2	120.52 (9)	O1—C8—H8A	110.2
C4—C3—H3	119.7	C9—C8—H8A	110.2
C2—C3—H3	119.7	H8B—C8—H8A	108.5
C5—C4—C3	120.27 (10)	C8—C9—H9A	109.5
C5—C4—H4	119.9	C8—C9—H9B	109.5
C3—C4—H4	119.9	H9A—C9—H9B	109.5
O2—C5—C4	118.65 (9)	C8—C9—H9C	109.5
O2—C5—C6	121.81 (9)	H9A—C9—H9C	109.5
C4—C5—C6	119.51 (9)	H9B—C9—H9C	109.5
O1—C6—C7	125.25 (9)	C1—N1—N1^i^	111.99 (10)
O1—C6—C5	114.69 (9)	C6—O1—C8	116.35 (8)
C7—C6—C5	120.07 (9)	C5—O2—H2	109.5
			
N1—C1—C2—C3	174.08 (10)	C4—C5—C6—C7	4.43 (15)
N1—C1—C2—C7	−5.05 (16)	O1—C6—C7—C2	177.64 (9)
C7—C2—C3—C4	2.56 (15)	C5—C6—C7—C2	−2.41 (15)
C1—C2—C3—C4	−176.60 (9)	C3—C2—C7—C6	−1.07 (15)
C2—C3—C4—C5	−0.53 (16)	C1—C2—C7—C6	178.05 (9)
C3—C4—C5—O2	179.09 (9)	C2—C1—N1—N1^i^	−178.00 (10)
C3—C4—C5—C6	−2.96 (15)	C7—C6—O1—C8	−7.68 (15)
O2—C5—C6—O1	2.27 (14)	C5—C6—O1—C8	172.36 (9)
C4—C5—C6—O1	−175.61 (9)	C9—C8—O1—C6	−177.35 (9)
O2—C5—C6—C7	−177.68 (9)		

Symmetry code: (i) −*x*+2, −*y*+1, −*z*+1.

### Hydrogen-bond geometry (Å, º)

**Table:**

*D*—H···*A*	*D*—H	H···*A*	*D*···*A*	*D*—H···*A*
O2—H2···N1^ii^	0.84	1.99	2.7787 (12)	156

Symmetry code: (ii) −*x*+3/2, *y*+1/2, −*z*+1/2.
